# Genome-wide identification of PEBP gene family in *Hedychium coronarium*

**DOI:** 10.3389/fpls.2025.1482764

**Published:** 2025-07-09

**Authors:** Qin Wang, Yiwei Zhou, Fang Wang, Xinyue Li, Yunyi Yu, Rangcai Yu, Yanping Fan

**Affiliations:** ^1^ The Research Center for Ornamental Plants, College of Horticulture, South China Agricultural University, Guangzhou, China; ^2^ Guangdong Provincial Key Laboratory of Ornamental Plant Germplasm Innovation and Utilization, Environmental Horticulture Research Institute, Guangdong Academy of Agricultural Sciences, Guangzhou, China; ^3^ College of Life Sciences, South China Agricultural University, Guangzhou, China

**Keywords:** *Hedychium coronarium*, PEBP gene family, flowering regulation, *HcPEBP11*, *expression patterns*

## Abstract

The Phosphatidylethanolamine-binding protein (PEBP) gene family plays a crucial role in plant growth and development, particularly in regulating flowering time and morphogenesis. However, the diversity, expression patterns, and functions of *PEBP* genes in *Hedychium coronarium* remain largely unexplored. In this study, 14 *HcPEBP* genes were identified and classified into MFT, FT, and TFL1 subfamilies based on phylogenetic analysis. Intraspecific collinearity analysis revealed three collinear relationships within the HcPEBP gene family. Interspecific collinearity analysis across *H. coronarium*, rice, wild banana, and pineapple highlighted the evolutionary significance of specific *PEBP* genes. Motifs DPDxP and GxHR are conserved in HcPEBPs, which are essential for anion-binding activity. At the same position in the C-terminus, FT-likes contain the xGxGGR motif, while TFL1-likes possesses the TAARRR motif. 64.3% of *HcPEBP* genes consist of four exons and three introns. Promoter regions of *HcPEBP* genes are enriched with light-responsive elements, suggesting a primary response to light signals. Expression patterns analysis by qRT-PCR showed that seven *FT*-like genes are predominantly expressed in leaves, with increased expression during the transition from vegetative to reproductive growth. *HcPEBP11*, a *FT*-like gene, is highly expressed in inflorescence buds. Overexpression of *HcPEBP11* in tobacco induced early flowering, confirming its role in promoting flowering. This study provides a foundation for further research on the biological functions of the PEBP gene family in *H. coronarium* and elucidates the role of *HcPEBP11* in flowering regulation.

## 1 Introduction

Phosphatidylethanolamine-binding proteins (PEBPs) possess evolutionarily conserved phosphatidylethanolamine-binding domains and are prevalent in both prokaryotes and eukaryotes (Dong et al., 2020; Li et al., 2009; Rautureau et al., 2009). In plants, PEBPs are pivotal in regulating floral transition, determining architecture, and influencing seed dormancy and germination, as well as tuber formation and sink-source allocation (Abelenda et al., 2019, 2009; Bi et al., 2016; Chen et al., 2018; Goto, 2005; Lee et al., 2023; Navarro et al., 2015; Yamaguchi et al., 2005; Zhang S. H, et al., 2021). The PEBP gene family is categorized into three subfamilies: Flowering Locus T-like (FT-like), TERMINAL FLOWER 1-like (TFL1-like), and MOTHER OF FT AND TFL1-like (MFT-like) (Karlgren et al., 2011). MFT-like is considered the ancestral form of FT-like and TFL1-like, present in both basal land plants and seed plants. In contrast, FT-like and TFL1-like are exclusive to seed plants, suggesting their emergence as a result of seed plant evolution (Hedman et al., 2009; Karlgren et al., 2011; Liu et al., 2016; Xu et al., 2022). Despite extensive sequence similarity among *PEBP* genes, their functions diverge significantly (Wang et al., 2015).

*MFT*-like genes are implicated in seed development and germination through their involvement in abscisic acid (ABA) and gibberellin (GA) signaling pathways (Vaistij et al., 2018; Xi et al., 2010; Xi and Yu, 2010; Yu et al., 2019). *FT*-like and *TFL1*-like genes are essential in regulating the timing of flowering and morphogenesis. In *Arabidopsis thaliana*, FT and TFL1 proteins share 60% sequence identity but exhibit antagonistic functions (Ahn et al., 2006; Hanzawa et al., 2005). The FT protein, acting as florigen, plays a crucial role in promoting flowering (Corbesier et al., 2007). The *FT* gene integrates both external and internal signals to regulate flowering (Abe et al., 2005; Wigge et al., 2005a). In rice, *Hd3* and *RFT1*, homologs of the *A. thaliana FT* gene, initiate flowering, also known as heading (Tamaki et al., 2007; Zhao et al., 2015). In maize, *ZCN8*, a *FT*-like gene, acts as a floral activator and contributes to photoperiod sensitivity. Its ectopic expression in vegetative shoot apices triggers early flowering in transgenic plants (Lazakis et al., 2011; Mach, 2011). *FT*-like genes, encoding florigens, have been identified in various plant species, including herbaceous plants, woody plants, and lianas (Fan et al., 2023; Igasaki et al., 2008; Kalia et al., 2023; Kim et al., 2022; Lembinen et al., 2023; Lin et al., 2019; J. Odipio et al., 2020; Patil et al., 2022). *TFL1*-like genes, comprising *TFL1*, *CEN* (*CENTRORADIALIS*), and *BFT* (*BROTHER OF FT*), act as floral repressors by delaying flowering and inhibiting flower primordia formation. TFL1 regulates shoot meristem identity and represses flowering by competitively binding to FD (a bZIP type transcription factor *FLOWERING LOCUS D*), thereby inhibiting FD-FT complex formation (Freytes et al., 2021; Kaneko-Suzuki et al., 2018; Zhu et al., 2020). In *Arabidopsis*, heterologous expression of apple (*Malus* × *domestica* Borkh) *MdTFL1* or *MdTFL2* significantly delays flowering, increases rosette leaf number, and plant height (Zuo et al., 2021). Mutations in the *TFL1* homologs *KSN* of rose and strawberry result in a continuous flowering habit (Iwata et al., 2012; Soufflet-Freslon et al., 2021). Additionally, in *Arabidopsis* and pea, *TFL1* modulates the length of the vegetative phase (Bradley et al., 1997; Foucher et al., 2003).

*PEBP* genes are integral to the growth, development, and reproduction of plants, making the analysis of their evolution and function are crucial for advancing plant cultivation and understanding. The PEBP gene family has been identified and researched in various plant species, including *A. thaliana* (Kardailsky et al., 1999), potato (Zhang et al., 2022), maize (Danilevskaya et al., 2008), rice (Zhao et al., 2022), *Dendrobium huoshanense* (Song et al., 2021), cotton (Wang et al., 2019; Zhang et al., 2016), *Perilla frutescens* (Xu et al., 2022), wheat (Dong et al., 2020), and tomato (Sun et al., 2023). However, data on the gene family’s member count, phylogeny, expression, and functions in *H. coronarium* remain unexplored. Known as “white ginger lily” or “butterfly ginger,” *H. coronarium* is a perennial herb and ornamental flowering plant native to the Eastern Himalayas and southern China (Baez et al., 2011). Its elegant floral morphology and refreshing scent have made it a popular choice for cultivation as a cut flower or garden plant in tropical and subtropical regions (Ke et al., 2019). Flowering, a critical agronomic trait for ornamental plants, affects their aesthetic and cultivation value. *H. coronarium* exhibits a continuous flowering habit, producing new aboveground stems and blooming from May to October.

This study conducted a genome-wide identification of the PEBP gene family in *H. coronarium*, revealing 14 *HcPEBP* genes. Using bioinformatics techniques, we analyzed sequence characteristics, phylogenetic relationships, chromosomal localization, conserved motifs, gene structures, collinearity, and *cis*-regulatory elements. qRT-PCR analysis provided insights into the expression patterns of *HcPEBPs* across various tissues. We specifically examined the expression level of *HcPEBP11* in leaf buds and inflorescence bud’s three distinct developmental stages. Notably, *HcPEBP11* exhibited higher expression in the inflorescence buds compared to other tissues. The role of *HcPEBP11* in promoting flowering was further investigated by its heterologous expression in tobacco. In summary, this work offers a scientific reference for understanding the PEBP gene family of *H. coronarium*. Additionally, this research lays the groundwork for further detailed investigations into the molecular and biological functions of HcPEBP gene family members and enriches the study of PEBP gene family in plant species.

## 2 Materials and methods

### 2.1 Plant material

*H. coronarium* was cultivated in the open field at the College of Horticulture, South China Agricultural University (Guangzhou, China, 23.16°N, 113.36°E). The plant materials (root, rhizome, leaf, leaf buds, inflorescence buds) were collected from 19:00 on June 15, 2024. Root, rhizome and leaf were collected during reproductive growth. All tissues were immediately frozen in liquid N and stored at − 80 until use.

### 2.2 PEBP gene family elucidation in *H. coronarium*

*Arabidopsis* PEBP sequences were downloaded from TAIR (https://www.arabidopsis.org/). The *H. coronarium* genome (unpublished) was provided by Beijing Novogene Bioinformatics Technology Corporation (China). *Arabidopsis* PEBP sequences were used as queries to identify HcPEBP sequences from the *H. coronarium* genome using the BLAST module in TBtools (E-value ≤ 1.0 × 10^-5^) (Chen et al., 2020). Additionally, The HMM profile of the PEBP domain (PF01161) from the Pfam database (http://pfam.xfam.org/) (Finn et al., 2006) was used in the Simple HMM search module in TBtools. Sequences identified by both BLAST and HMM searches were merged, and duplicates were removed. All candidate sequences were analyzed for the PEBP domain using the NCBI-Conserved Domain Database (https://www.ncbi.nlm.nih.gov/Structure/cdd/wrpsb.cgi) (Morris et al., 2015). Only sequences containing the complete PEBP domain were retained for further analysis.

### 2.3 Chromosomal location and collinearity analysis

Chromosomal details (length, gene density, and gene positions) were extracted from the *H. coronarium* genome using TBtools (Chen et al., 2020). The chromosomal locations of *HcPEBP* genes were visualized using TBtools. Genome sequences and annotations for wild banana, pineapple, and rice were downloaded from NCBI (https://www.ncbi.nlm.nih.gov/) and Ensemble Plants (https://plants.ensembl.org). Gene duplication events and collinear relationships were analyzed using the One-step MCScanX-Super Fast module in TBtools with default settings. Collinear relationships were visualized using the Dual Synteny Plotter in TBtools.

### 2.4 Conserved motif and gene structure analysis

Conserved motifs within HcPEBP proteins were analyzed using the MEME suite (https://meme-suite.org/meme/tools/meme) (Bailey et al., 2015). The motif count was set to eight, with other parameters kept at default settings. The identified motifs and gene structures were visualized using the Gene Structure View (advanced) module in TBtools.

### 2.5 Investigation of physicochemical characteristics

Physicochemical properties of HcPEBP proteins, including molecular weight (MW), isoelectric point (pI), instability index, aliphatic index, and GRAVY (grand average of hydropathicity), were analyzed using the ProtParam tool on the ExPASy online platform (http://www.expasy.ch/tools-/pi_tool.html) (Artimo et al., 2012). Subcellular localization predictions for HcPEBP proteins were made using the WoLF PSORT tool (Horton et al., 2007).

### 2.6 Alignment of multiple sequences and evolutionary relationship study

PEBP protein sequences from *Arabidopsis* and rice were downloaded from TAIR and the Rice Genome Annotation Project (RGAP, http://rice.uga.edu/) databases, respectively. The GenBank accession numbers for PEBPs in *Arabidopsis* and rice are listed in **Supplementary Table S1**. Multiple sequence alignments of PEBP proteins from *H. coronarium*, *Arabidopsis*, and *Oryza sativa* were conducted using TBtools. A phylogenetic tree comprising 40 PEBP proteins from *H. coronarium*, *Arabidopsis*, and rice was constructed with TBtools using default parameters and visualized with Evolview (https://www.evolgenius.info/evolview-v2) (Zhang et al., 2012). Amino acid sequences of HcPEBPs were aligned using the ClustalW algorithm in MEGA11 and displayed using GeneDoc (Tamura et al., 2021).

### 2.7 Examination of *cis*-regulatory elements

Sequences 2000 bp upstream of the transcription start site (ATG) for *HcPEBP* genes were extracted from the genome sequence using TBtools. *Cis*-acting elements within the promoter sequences of *HcPEBPs* were predicted using the PlantCARE database (http://bioinformatics.psb.ugent.be/webtools/plantcare/html/) (Lescot et al., 2002), with results analyzed, classified, and visualized using TBtools.

### 2.8 Differential gene expression profiling across various tissues

The expression patterns of *HcPEBP* genes across various tissues of *H. coronarium* were investigated. Relative expression levels in five tissues (root, rhizome, leaf, inflorescence bud, leaf bud) were measured by qRT-PCR. Morphological characteristics of these tissues are depicted in **Supplementary Figure S1**. Primer sequences are provided in **Supplementary Table S2**.

### 2.9 Total RNA and DNA extraction, cDNA synthesis and qRT-PCR analysis

Total RNA was isolated from plant materials using the HiPure Plant RNA Mini Kit (Magen, Guangzhou, China), following the manufacturer’s protocol. DNA was extracted using the DNA Quick Plant System (Tian Gen, Beijing, China), as per the provided manual. For gene cloning, cDNA was synthesized using the PrimeScript™ RT Reagent Kit with gDNA Eraser (TaKaRa, Japan). For qRT-PCR, cDNA was reverse-transcribed using the Evo M-MLV RT Mix Kit with gDNA Clean for qPCR Ver.2 (Accurate Biology, Hunan, China). qRT-PCR was performed using Hieff^®^ qPCR SYBR Green Master Mix (Yeasen Biotechnology, Shanghai, China) on an ABI 7500 Fast Real-Time PCR system (Applied Biosystems, USA). Reaction conditions followed a previously described protocol (Wang et al., 2021). The GAPDH gene was used as an internal reference for normalization. Relative gene expression levels were calculated using the 2−ΔΔCt method. Statistical analyses for Significant differences were determined using IBM SPSS Statistics and Origin 2021.

### 2.10 Molecular cloning and genetic engineering in plants

The coding sequence (CDS) of *HcPEBP11* was amplified from *H. coronarium* cDNA using Phanta Max Super-Fidelity DNA Polymerase (Vazyme, Nanjing, China), following the manufacturer’s protocol. The CDS was cloned into the pOx vector (provided by the State Key Laboratory for Conservation and Utilization of Subtropical Agro-Bioresources, South China Agricultural University, China) using the ClonExpress II One Step Cloning Kit (Vazyme, Nanjing, China). The constructed plasmid was transformed into Agrobacterium tumefaciens GV3101 (WeDi, Shanghai, China) using the freeze-thaw method. Primers used are listed in **Supplementary Table S2**. Tobacco plants (*Nicotiana tabacum* cv. W38) were transformed using the leaf disc method (Horsch et al., 1986). Agrobacterium cultures containing *pOx-HcPEBP11* were grown at 28°C in liquid medium with 50 µg/mL kanamycin and 25 µg/mL rifampicin until reaching an OD600 of 0.6–0.8. Bacteria were pelleted by centrifugation at 5000 rpm for 8 minutes and resuspended in MS liquid medium containing 100 µM acetosyringone to an OD600 of 0.7–0.8. Young leaf explants from sterile tobacco seedlings were pre-cultured for three days in darkness before transformation. Transformed plants were regenerated through a series of cultures: co-culture, bacteriostatic, differentiation, induction, rooting, and screening.

### 2.11 Selection and characterization of transgenic lines with phenotypic evaluation

Putative transgenic tobacco plants resistant to 20 mg/L hygromycin B in 1/2 MS medium were screened using PCR (Cordero Otero and Gaillardin, 1996; Thion et al., 2002; Walter et al., 1989). Universal primers pOx-F/R (flanking the multiple cloning sites of the pOx vector) and specific primers HPH-F/R (targeting the hygromycin B phosphotransferase gene) were used to confirm the integration of *HcPEBP11* into the tobacco genome. Primer sequences are listed in **Supplementary Table S2**. Semi-quantitative RT-PCR (Semi-qRT-PCR) and qRT-PCR were performed to verify *HcPEBP11* expression in transgenic lines (Tripathi et al., 2022). Total RNA was extracted from leaves at the flower bud stage. Semi-qRT-PCR was conducted using ABI VeritiPro PCR (Thermo Fisher) and Phanta Max Super-Fidelity DNA Polymerase, with specific primers for *HcPEBP11*. Transgenic and wild-type tobacco lines were grown under natural light conditions. The time to bolting (appearance of the first flower bud) and flowering (blooming of the first flower) was recorded to assess the role of *HcPEBP11* in flowering regulation.

### 2.12 Subcellular localization of HcPEBP11 protein

The coding sequence (CDS) of the *HcPEBP11* gene, lacking termination codons, was successfully cloned into the p35S-cGFP vector. Subsequently, the resultant recombinant vector underwent transformation into the *A. tumefaciens* strain GV3101 via the heat shock method. For the transformation assay, leaves of *N. benthamiana* at the five-leaf stage were utilized (Kokkirala et al., 2010). Post 72 hours of infiltration, the green fluorescent protein (GFP) signals were detected using a confocal laser scanning microscope.

## 3 Results

### 3.1 Identification and physicochemical characterization of HcPEBP gene family

In *H. coronarium*, 14 *PEBP* genes were identified and named *HcPEBP1–14* based on their chromosomal distribution. Key details about the *HcPEBP* family and the physicochemical properties of their encoded proteins are summarized in **Table 1** and **Supplementary Table S3**. Protein length ranges from 142 aa (HcPEBP7) to 201 aa (HcPEBP14), with an average of 175 aa. Molecular weight averages 19.71 kDa, ranging from 15.97 kDa (HcPEBP7) to 22.90 kDa (HcPEBP14). Theoretical isoelectric points span from 5.93 (HcPEBP11) to 11.28 (HcPEBP7). Instability Index ranges from 34.77 (HcPEBP4) to 61.06 (HcPEBP13). Aliphatic index aries between 87.73 (HcPEBP8) and 72.92 (HcPEBP13). All 14 HcPEBP proteins are hydrophilic. Except for HcPEBP13 (mitochondrial), all other HcPEBPs are predicted to be cytoplasmic proteins.

**Table 1:** HcPEBP gene family protein properties table.

Gene name	Chr	Position (5’-3’)	CDS length (bp)	Protein Length (aa)	Protein characteristics					
					MW (kDa)	Theoretical pI	Instability index	Aliphatic index	GRAVY	Subcellular Location
*HcPEBP1*	2	36994679-36995807	546	181	20.3	9.09	42.8	82.82	-0.366	Cyto
*HcPEBP2*	2	53803737-53804784	525	174	19.49	9.03	43.03	78.33	-0.23	Cyto
*HcPEBP3*	3	1899620-1900405	465	154	17.61	8.55	42.1	76.49	-0.277	Cyto
*HcPEBP4*	3	6097205-6098271	528	175	19.93	7.79	34.77	73.37	-0.447	Cyto
*HcPEBP5*	5	48908632-48909372	531	176	19.48	9	35.32	79.77	-0.282	Cyto
*HcPEBP6*	10	12422385-12423467	534	177	20.17	7.81	40.43	80.23	-0.345	Cyto
*HcPEBP7*	11	48845892-48846868	429	142	15.97	11.28	53.83	84.37	-0.131	Cyto
*HcPEBP8*	14	3018653-3019853	519	172	18.67	7.91	45.14	87.73	-0.048	Cyto
*HcPEBP9*	14	41829107-41831720	543	180	20.27	8.82	36.59	76.28	-0.281	Cyto
*HcPEBP10*	Scaffold:000248F	294205-295281	534	177	19.87	7.89	43.74	76.44	-0.308	Cyto
*HcPEBP11*	Scaffold: 000248F	303025-307198	537	178	20.04	5.93	40.1	85.28	-0.176	Cyto
*HcPEBP12*	Scaffold: 000263F	970783-971610	534	177	20.05	8.73	36.19	79.21	-0.287	Cyto
*HcPEBP13*	Scaffold: 000345F	895323-896173	588	195	21.15	9.8	61.06	72.92	-0.24	Mito
*HcPEBP14*	Scaffold: 000371F	4752-7052	606	201	22.9	7.75	37.51	84.28	-0.278	Cyto

### 3.2 Chromosomal localization and gene duplication analysis of *HcPEBP* genes

**Figure 1:**
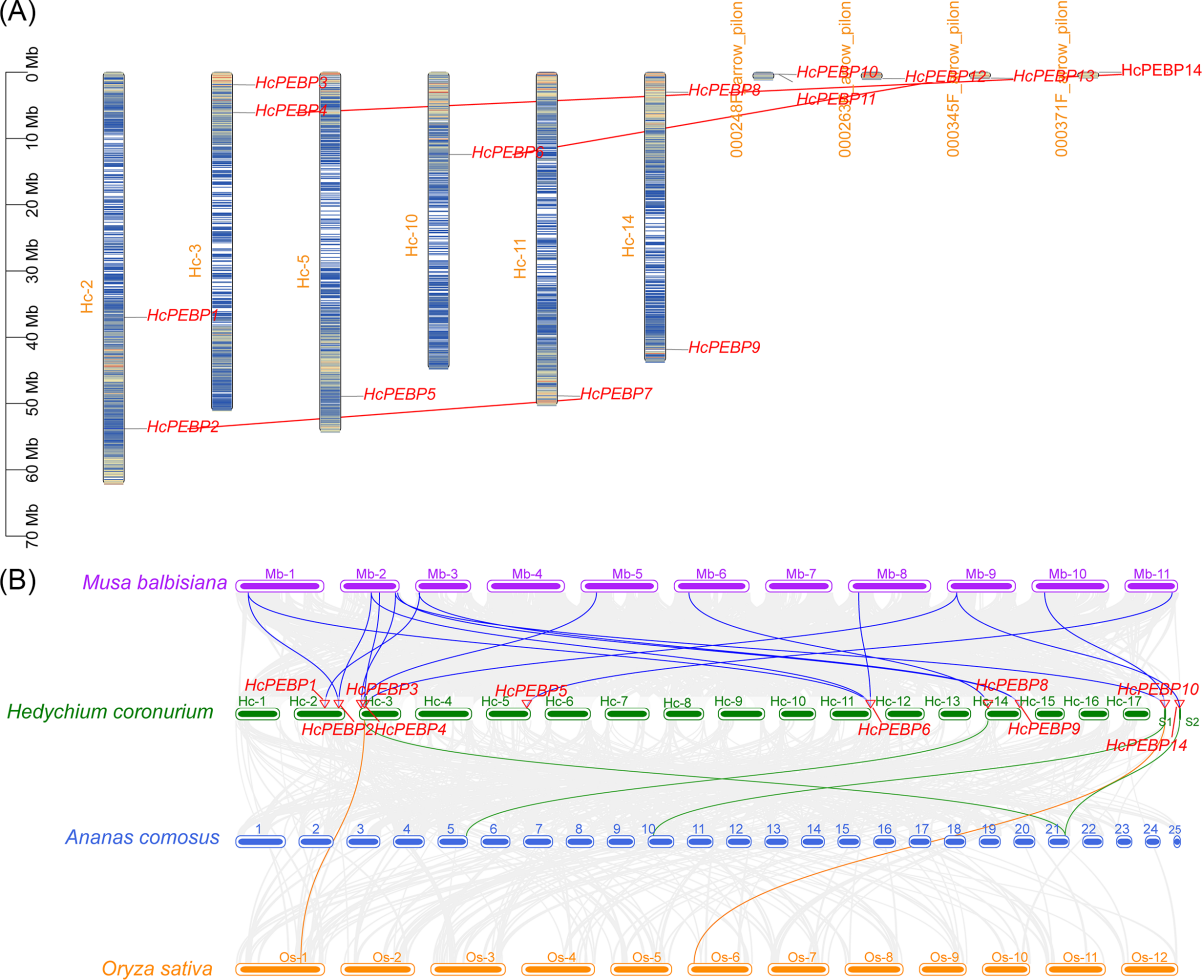
Chromosomal localization and collinearity analysis of *HcPEBP* genes. **(A)** Chromosome length is shown on the left scale. *HcPEBP* genes (1–14) are highlighted in red; chromosome numbers are in orange. The gene density is indicated by a blue-to-red gradient. Red lines show intraspecific collinearity among *HcPEBP* genes. **(B)** Interspecific collinearity analysis of *HcPEBP* genes with *M. balbisiana*, *A*. *comosus*, and *O. sativa*. Chromosome numbers are displayed above each chromosome. Colored lines indicate collinear relationships; red triangles mark *HcPEBP* gene locations.

In *H. coronarium*, 14 *HcPEBP* genes were identified. 9 genes (*HcPEBP1-9*) mapped to six chromosomes (Hc-2, Hc-3, Hc-5, Hc-10, Hc-11, Hc-14), while the remaining five genes (*HcPEBP10-14*) are located on four genome scaffolds. Intraspecific collinearity analysis revealed three pairs of duplicated genes: *HcPEBP2* and *HcPEBP7*, *HcPEBP4* and *HcPEBP14*, *HcPEBP6* and *HcPEBP12* (**Figure 1A**). To assess the impact of selection pressure on the collinear HcPEBP gene pairs, the Ka/Ks ratios (non-synonymous to synonymous substitutions) were calculated for each duplicated pair. A Ka/Ks = 1 indicates neutral selection, Ka/Ks < 1 indicates purifying selection, and Ka/Ks > 1 suggests positive selection. The Ka/Ks ratios for the three collinear *HcPEBP* gene pairs ranged from 0.087 to 0.295 (**Supplementary Table S4**), indicating that these duplicated gene pairs have undergone purifying selection, which maintains functional stability by removing deleterious mutations.

To further explore the evolutionary relationships of *HcPEBP* genes with those of other species, a collinearity analysis was conducted using *O. sativa*, *Musa balbisiana*, and *Ananas comosus* alongside HcPEBP family members. The results revealed that the HcPEBP gene family exhibits 17 collinearities with wild banana, 2 with rice, and 4 with pineapple (**Figure 1B**, **Supplementary Table S5**). Among these species, *HcPEBP* genes show the closest evolutionary relationship with PEBP members from wild banana. Notably, *HcPEBP4* and *HcPEBP10* display collinearities with all three species, highlighting their conserved evolutionary roles.

### 3.3 Phylogenetic analysis of PEBP family members

**Figure 2:**
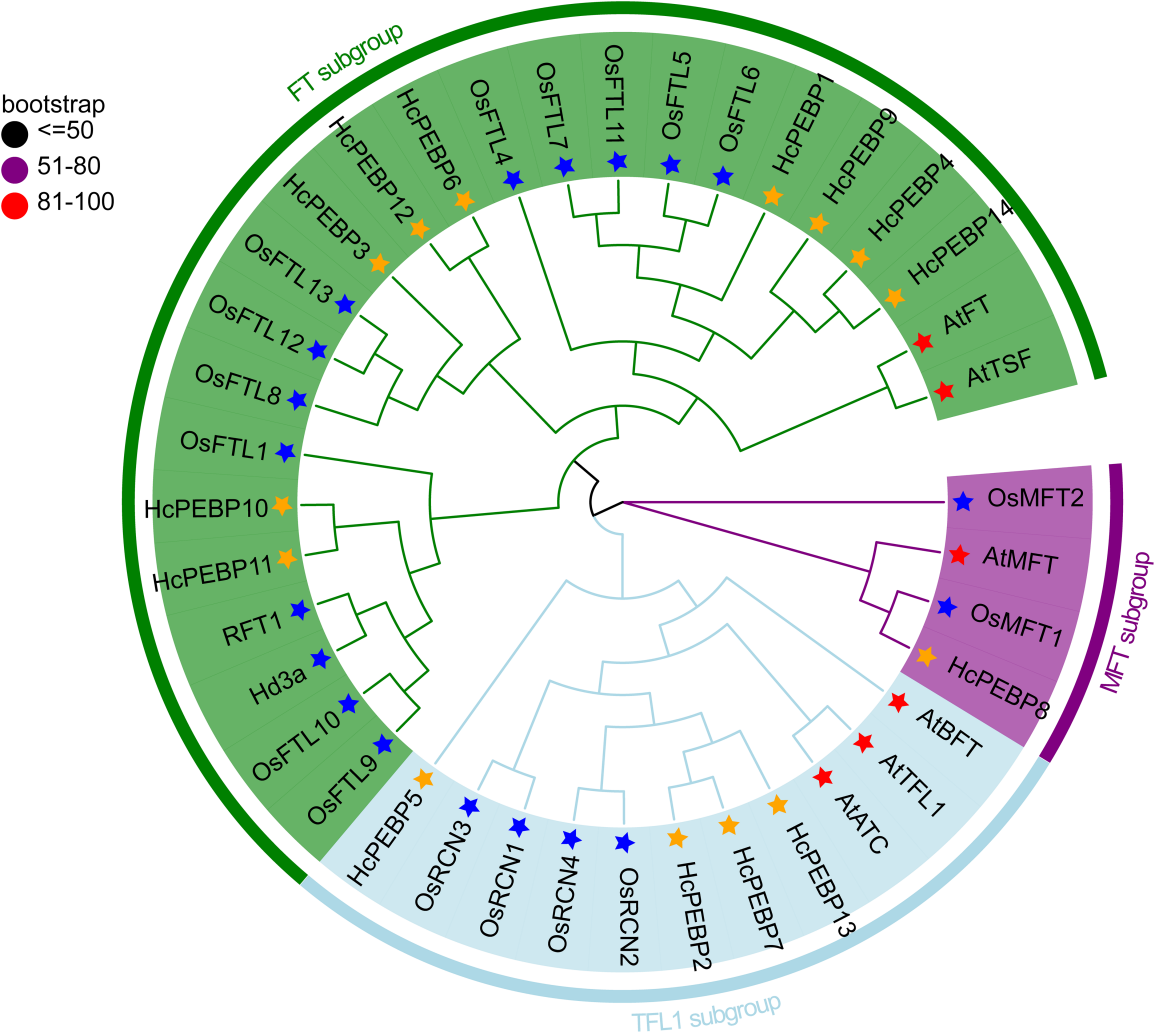
Phylogenetic tree analysis of 6 AtPEBP (*A. thaliana*), 20 OsPEBP (*O. sativa*), and 14 HcPEBP (*H. coronarium*) proteins. Species marked by colored stars: Red: *A. thaliana*, Blue: *O. sativa*, Orange: *H. coronarium.* Bootstrap percentage values (1,000 replications) at nodes: Black circles (0–0.5), Purple circles (0.51–0.8), Red circles (0.81–1.0). GenBank accession numbers for AtPEBPs and OsPEBPs are provided in **Supplementary Table S1**.

To investigate the evolutionary relationships of *HcPEBP* genes with *PEBP* genes from other species, a phylogenetic tree was constructed using multiple sequence alignments of amino acids from 6 PEBP proteins of *A. thaliana*, 20 PEBP proteins of *O. sativa*, and 14 PEBP proteins of *H. coronarium* (**Figure 2**). The phylogenetic tree is divided into three subgroups: MFT, TFL1, and FT. *HcPEBP8* clusters with *AtMFT*, *OsMFT1*, and *OsMFT2*, indicating it belongs to the MFT subgroup. Four genes (*HcPEBP2/5/7/13*) cluster with *AtBFT*, *AtTFL1*, *AtATC*, and *OsRCEs*, suggesting these four genes belong to the TFL1 subgroup. Nine genes (*HcPEBP1/3/4/6/9/10/11/12/14*) cluster with *AtFT*, *AtTSF*, *Hd3a*, and *OsFTLs*, indicating they belong to the FT subgroup.

### 3.4 Alignment of amino acid sequences and analysis of conserved domains

**Figure 3:**
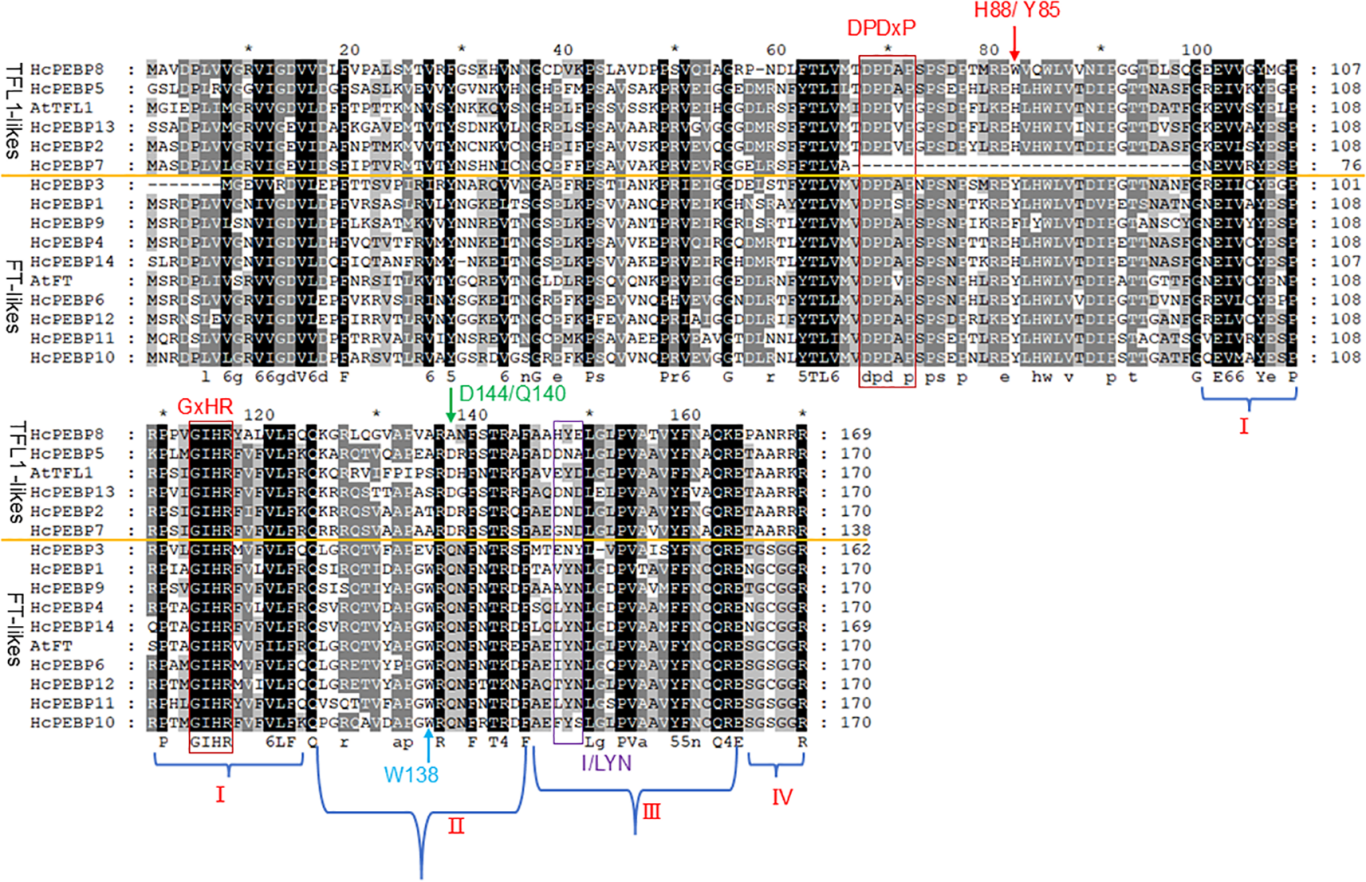
Multiple sequence alignment of HcPEBPs. AtFT and AtTFL1.

Phylogenetic tree analysis revealed that *HcPEBP1/3/4/6/9/10/11/12/14* belong to the FT subfamily, while *HcPEBP2/5/7/13* belong to the TFL1 subfamily. To further investigate the structural and functional conservation of these proteins, amino acid sequence alignment was performed for HcPEBPs, along with AtFT and AtTFL1 (**Figure 3**). All HcPEBP proteins contain the GxHR motif, a hallmark of the PEBP family. Except for HcPEBP7, all other HcPEBP proteins possess the DPDxP motif, another key functional domain. The C-terminal amino acid sequence can be divided into four segments (I–IV), each of which is essential for the FT protein’s function. The conserved motif LYN/IYN/in FT-like proteins is located in segment III, playing a critical role in enzymatic activity. In segment IV region, FT-like proteins contain the motif xGxGGR, whereas TFL1-like proteins contain a different motif (TAARRR) in the same region. FT-like proteins HcPEBP1/3/6/10/11/12 contain the key amino acid residues Tyr85, Trp138, and Gln140, which are critical for their function. In contrast, TFL1-like proteins HcPEBP2/5/13 possess the key residues His88 and Asp144, which are characteristic of their functional role. Interestingly, in FT-like proteins HcPEBP4/9/14, the residue Tyr85 is mutated to His, Phe, His, respectively.

### 3.5 Analysis of conserved motifs and gene structural features

**Figure 4:**
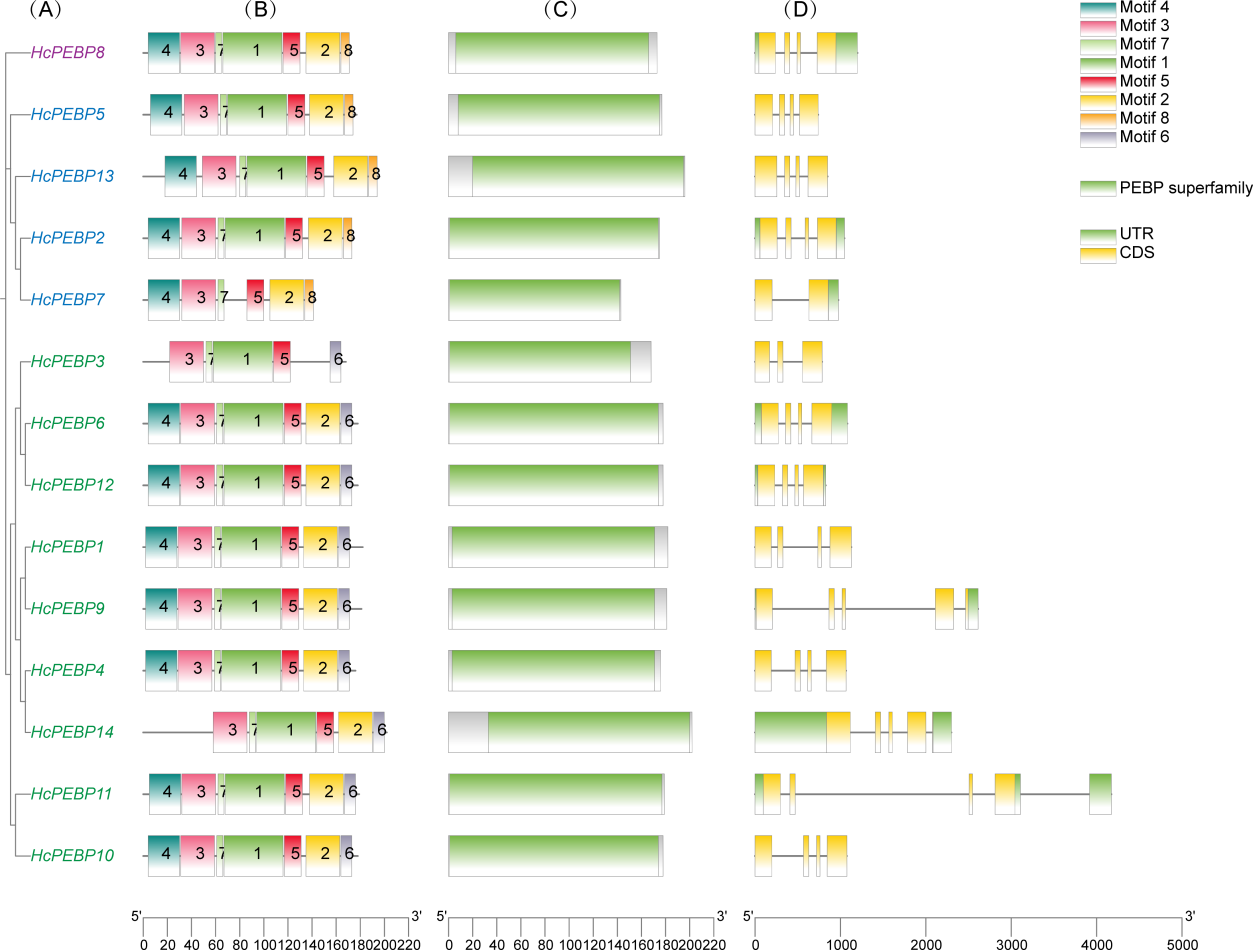
Phylogenetic tree, conserved motifs, domain, and gene structure analysis of HcPEBP gene family members. **(A)** Phylogenetic tree. **(B)** Distribution of conserved motifs in HcPEBP proteins, represented by colored boxes. **(C)** Conserved domains of HcPEBP proteins. **(D)** Gene structure of HcPEBP family members, showing UTRs (green rectangles), exons (yellow rectangles), and introns (grey lines). A scale at the bottom allows comparison of protein and gene lengths.

A phylogenetic tree containing only *HcPEBP* genes was constructed, dividing them into three subfamilies (**Figure 4A**). To further investigate the characteristics of *HcPEBP* genes, we conducted conserved motif and gene structure analyses. In the conserved motif analysis (**Figure 4B**), 8 conserved motifs (named motifs 1–8) were identified, with detailed sequence information provided in **Supplementary Table S6**. The analysis revealed the following patterns: Motifs 3, 5, and 7 are shared by all HcPEBPs, indicating their fundamental role in the protein family. Motif 1 is present in all HcPEBPs except HcPEBP7. Motif 2 is found in all HcPEBPs except HcPEBP3. Motif 4 is absent in HcPEBP3 and HcPEBP14. Motif 6 is exclusively present in the FT subfamily, highlighting its specificity to this group. Motif 8 is unique to the TFL1 and MFT subfamilies, suggesting a functional role specific to these groups. Additionally, conserved domain analysis confirmed that all 14 HcPEBPs contain the PEBP domain (**Figure 4C**).

Gene structure analysis revealed that the 14 *HcPEBP* genes share a generally similar structural organization (**Figure 4D**). Among them: nine *HcPEBP* genes (64.3% of the total) contain four exons and three introns, representing the most common structural pattern in the HcPEBP gene family. Three *HcPEBP* genes (*HcPEBP9/11/14*) consist of five exons and four introns. *HcPEBP3* with three exons and two introns. *HcPEBP7* contain only two exons and one intron.

### 3.6 Prediction and characterization of *cis*-regulatory elements

**Figure 5:**
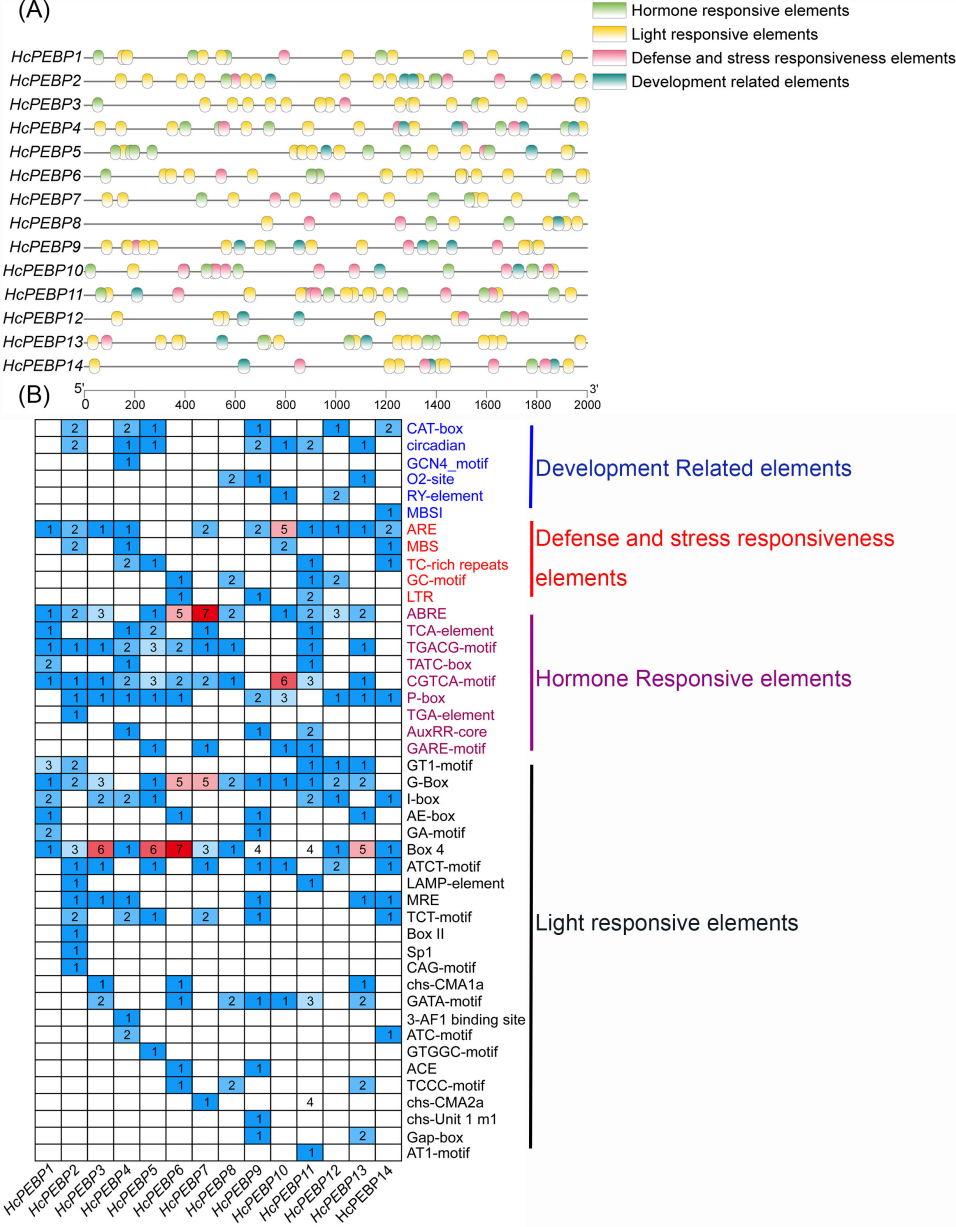
*Cis*-acting element analysis of *HcPEBP* gene promoter regions. **(A)** Distribution of *cis*-acting elements in the 2000 bp upstream promoter regions of *HcPEBP* genes. Different colors represent various element types. A ruler indicates sequence direction and length. **(B)** Classification and statistics of *cis*-acting elements. Numbers and colors indicate the count of specific elements per gene.

*Cis*-acting elements are critical for regulating gene transcription. In this study, we analyzed the 2000 bp upstream sequences of the *HcPEBP* genes’ start codon to predict and characterize *cis*-acting elements (**Figure 5A**). A total of 45 distinct *cis*-acting elements were identified and classified into four functional categories (**Figure 5B**): hormone-responsive (98 elements), development-related (28 elements), light-responsive (161 elements), and defense and stress responsiveness-related (40 elements), summing up to 327 elements. Among these, light-responsive elements were the most abundant, while development-related element were the least frequent. All *HcPEBP* genes, except *HcPEBP4* and *HcPEBP14*, contain the G-box element (light-Responsive Element). All genes, except *HcPEBP10*, contain the Box4 element. *HcPEBP1/3/6/7* lack any development-related elements. Detailed information on the *cis*-acting elements in the promoter regions of *HcPEBP* genes is provided in **Supplementary Table S7**.

### 3.7 Tissue-specific expression profiling of *HcPEBP* genes

**Figure 6:**
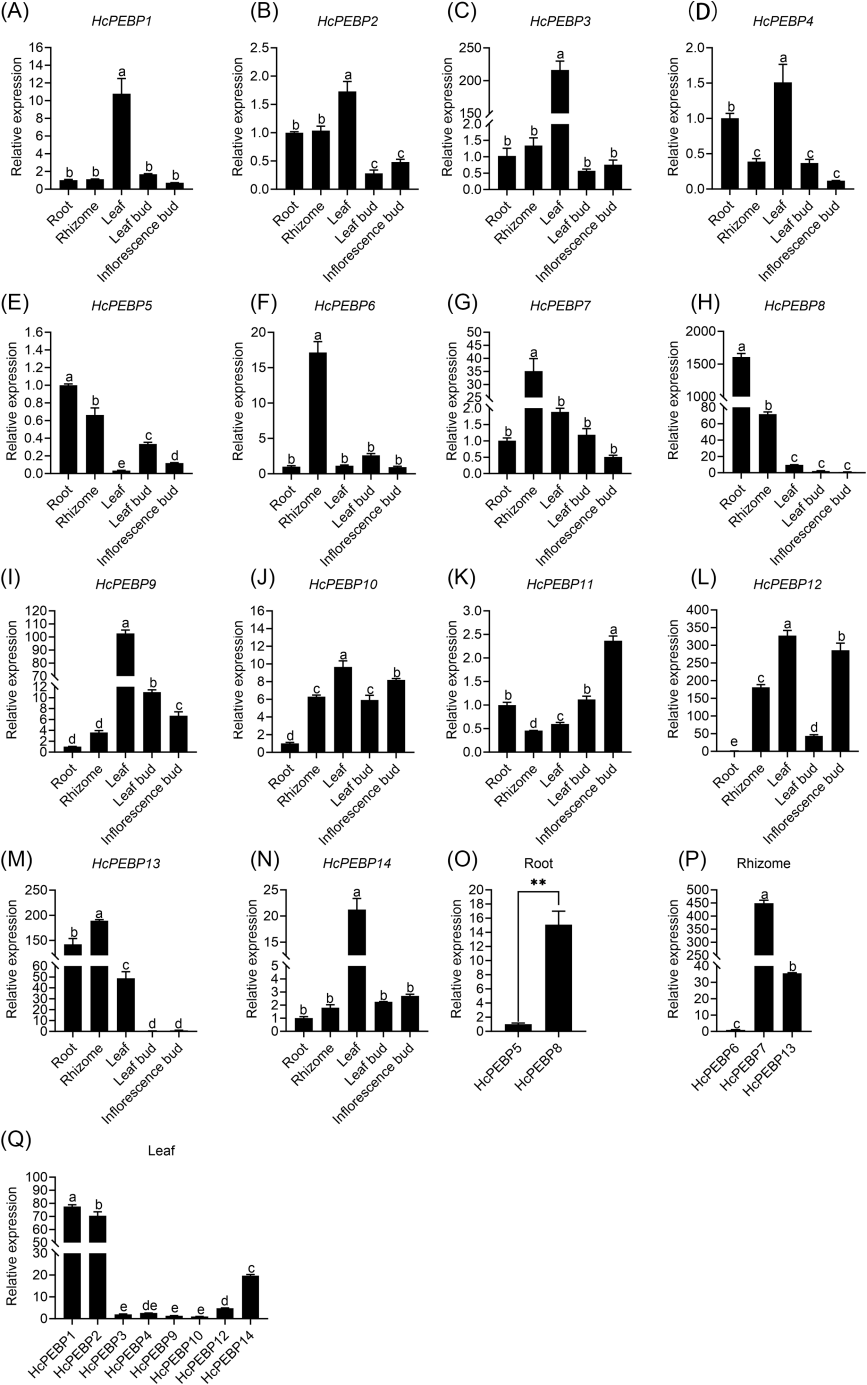
Tissue-specific expression analysis of *HcPEBP* genes. **(A–N)** Relative expression levels of *HcPEBP* genes in five tissues. **(O)** HcPEBP genes predominantly expressed in roots. **(P)** HcPEBP genes predominantly expressed in rhizomes. **(Q)** HcPEBP genes predominantly expressed in leaves. Error bars represent standard deviation (three biological replicates). Different lowercase letters indicate significant differences at *P*< 0.05 (after multiple comparison corrections). ** denotes *P*< 0.01.

**Figure 7:**
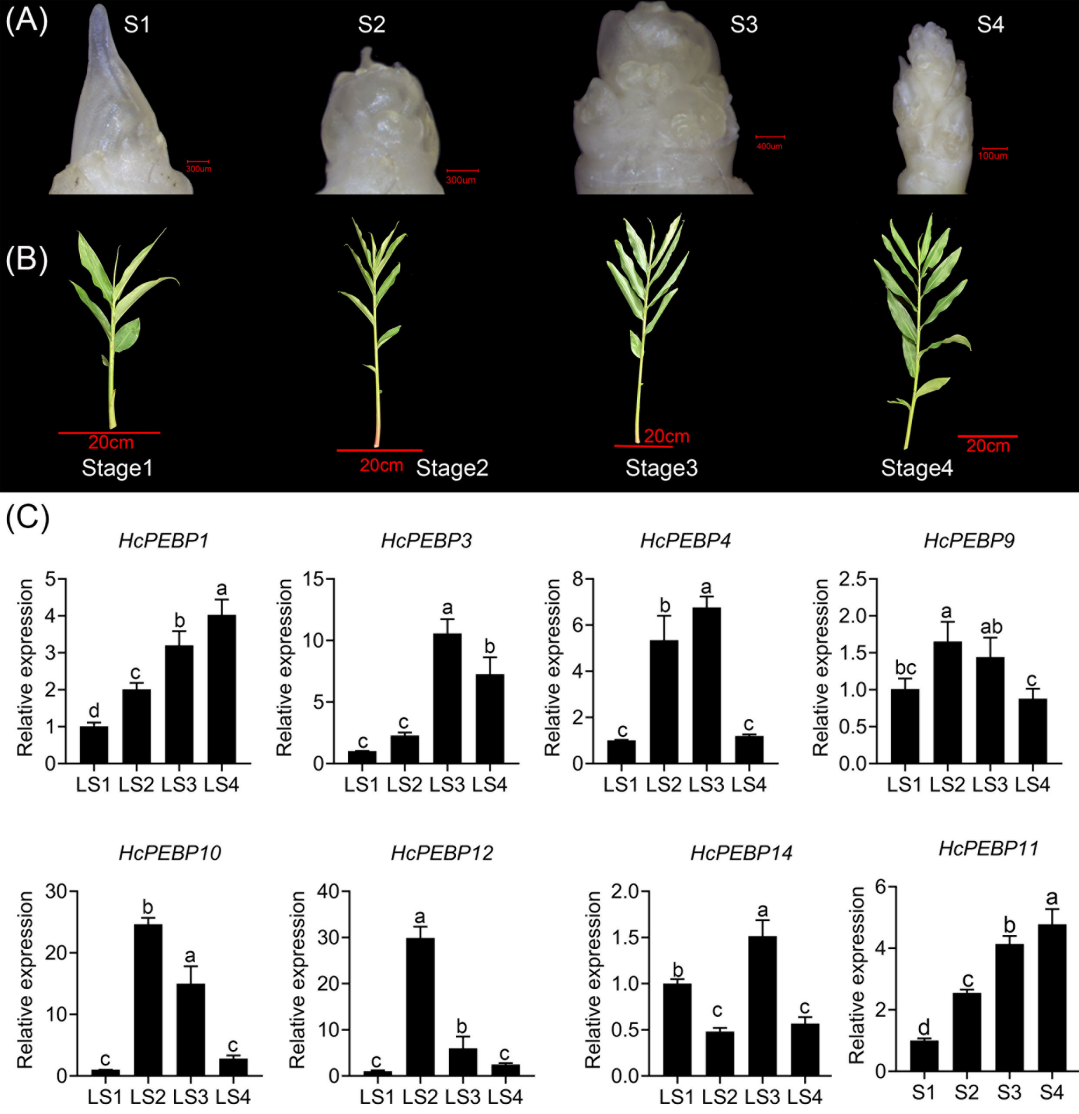
Quantitative expression analysis of eight FT subfamily *HcPEBP* genes. **(A)** Developmental stages of apical meristem in this study: S1, leaf bud. S2/3/4, early stage/middle stage/late stage of inflorescence bud differentiation. **(B)** Plants corresponding to apical meristem stages. **(C)** Expression levels of seven FT subfamily *HcPEBP* genes in leaves at four apical meristem stages and *HcPEBP11* during apical meristem development. ‘LSs’ denotes leaves at different apical meristem stages.

To investigate tissue-specific gene expression patterns, we analyzed the expression levels of *HcPEBP* genes across various tissues—root, rhizome, leaf, leaf bud, and inflorescence bud using qRT-PCR (**Supplementary Figure S1**). *HcPEBP5* and *HcPEBP8* showed the highest expression in roots, with *HcPEBP8* significantly exceeding *HcPEBP5* (**Figures 6E, H, O**). *HcPEBP1/2/3/4/9/10/12* and *HcPEBP14* exhibited significantly elevated expression in leaves compared to other tissues (**Figures 6A–D, I, J, L, N**). Among these, *HcPEBP1/2/14* had notably higher expression levels (**Figure 6Q**). *HcPEBP6/7/13* were most highly expressed in Rhizomes, with *HcPEBP7* levels significantly surpassing those of *HcPEBP6* and *HcPEBP13* (**Figures 6F–G, M, P**). *HcPEBP11* expression was significantly higher in inflorescence buds compared to other tissues (**Figure 6K**). Additionally, transcriptome data and qRT-PCR results indicate that the expression of *HcPEBP11* progressively increases during the development of inflorescence buds. (**Figures 7A, C**, **Supplementary Figure S2**, **Supplementary Table S9**). Seven FT subfamily genes (*HcPEBP1/3/4/9/10/12/14*) exhibited high expression levels in leaves (**Figures 6A, C, D, I, J, L, N**). To further investigate their roles, we analyzed their expression patterns in leaves at four developmental stages of the apical meristem (**Figures 7B, C**). The results revealed that *HcPEBP1* showed a continuous increase in expression from stage 1 to stage 4. *HcPEBP3/4/9/10/12* displayed an initial increase followed by a decrease, with *HcPEBP9/10/12* peaking at stage 2 and *HcPEBP3/4* reaching their highest expression at stage 3. These findings suggest that *HcPEBP1/9/10/12* may play roles in floral transition.

### 3.8 Functional verification of heterologous overexpression of *HcPEBP11* and subcellular localization of HcPEBP11

**Figure 8:**
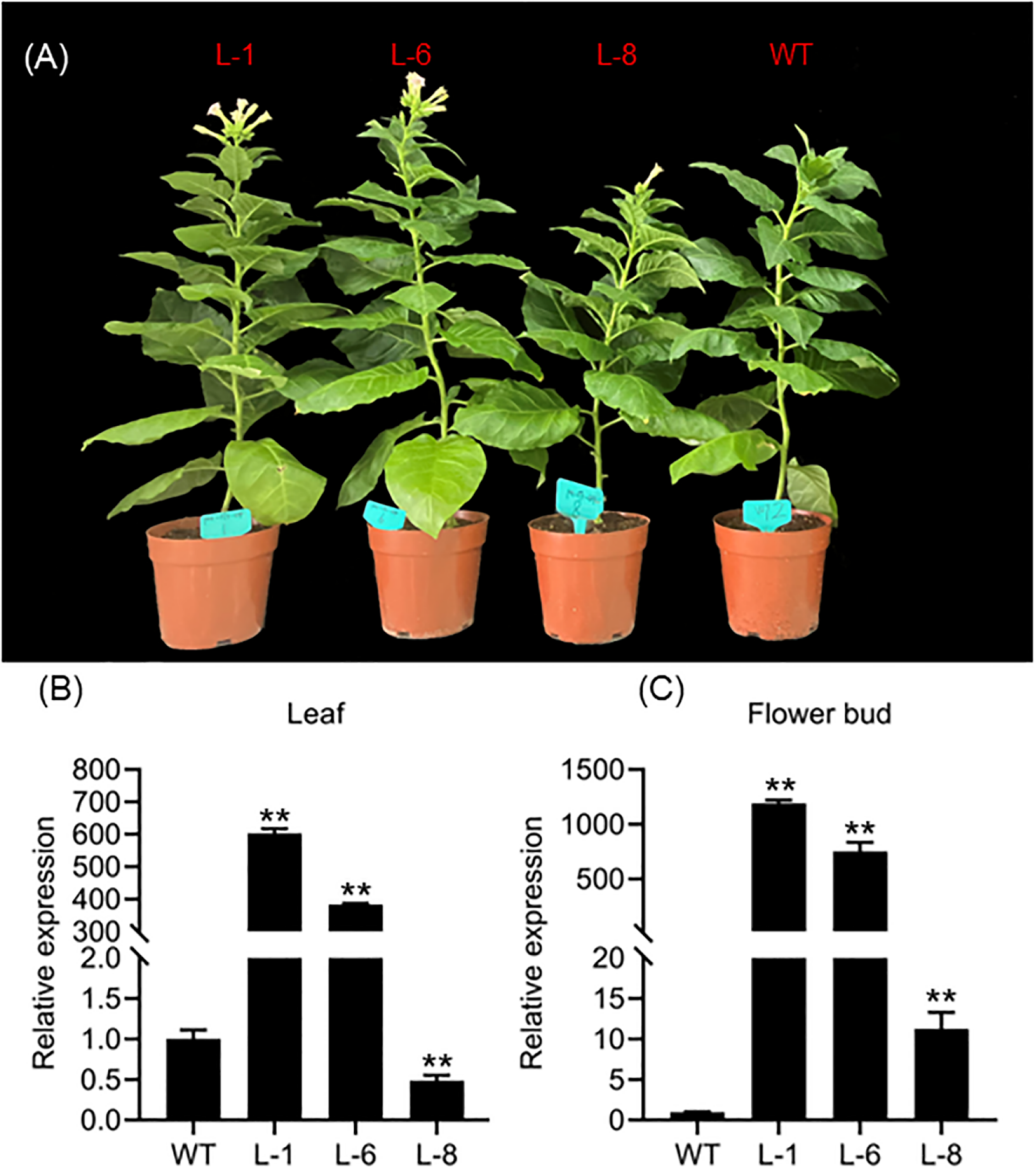
Flowering phenotype and *HcPEBP11* expression analysis in transgenic *tobacco.* **(A)** Flowering traits comparison between *HcPEBP11* transgenic *tobacco* and wild-type (WT) plants. ‘M’: Marker D2000, ‘WT’: Wild-type (negative control), ‘P’: *pOx*-*PEBP11* plasmid (positive control). L-1, L-6, L-8: *HcPEBP11* transgenic lines. **(B)** *HcPEBP11* expression levels in leaves of transgenic *tobacco*. **(C)** *HcPEBP11* expression levels in flower buds of transgenic *tobacco*. ** indicates *P* < 0.01.

Expression analysis of the HcPEBP gene family suggests that *HcPEBP11* may influence the timing of flowering. To elucidate *HcPEBP11*’s regulatory role in flowering, we overexpressed it in tobacco. PCR amplification confirmed the presence of *HcPEBP11* in transgenic tobacco strains and the positive control, with consistent band positions, while the negative control displayed no bands (**Supplementary Figures S3A, B**). This indicates the successful integration of *HcPEBP11* into the tobacco genome. Semi-quantitative PCR results revealed *HcPEBP11* expression in all transgenic lines, absent in wild type (WT) (**Supplementary Figure S3D**), confirming its expression in the transgenic strains. Expression analysis showed that *HcPEBP11* levels in the leaves and flower buds of transgenic strains L-1 and L-6 were significantly higher than in WT, with transgenic line L-8 exhibiting even higher levels in the flower bud (**Figures 8B, C**). Transgenic plants flowered earlier than WT, with bolting and flowering times occurring 9–11 and 8–12 days earlier, respectively (**Figure 8A**; **Supplementary Table S8**). Bioinformatic prediction and GFP assay validation indicated that *HcPEBP11* proteins localize to the cytoplasm (**Supplementary Table S4**; **Supplementary Figure S4**). These findings suggest that *HcPEBP11* promotes early flowering in tobacco and may function as a cytoplasmic regulator involved in flowering time control.

## 4 Discussion

PEBP proteins constitute a class of proteins characterized by a conserved PEBP domain. Extensive research has demonstrated that PEBPs perform a conserved role in regulating plant growth and developmental processes (Chardon and Damerval, 2005; Danilevskaya et al., 2008; He et al., 2022). The PEBP gene family is categorized into three subfamilies—FT-like, TFL1-like, and MFT-like—based on phylogenetic relationships. The FT and TFL1 subfamilies are instrumental in regulating flowering time, morphogenesis, and plant architecture (Xi and Yu, 2009), while the MFT subfamily is involved in seed development, germination, and dormancy (Chen et al., 2018; Footitt et al., 2017). Although the identification and functional characterization of the PEBP gene family have been extensively studied in *Arabidopsis* and other angiosperms (He et al., 2022; Li et al., 2023; Song et al., 2021; Sun et al., 2023; Venail et al., 2022; Xu et al., 2022; Yang et al., 2023; Zhang et al., 2022; Zhang M, et al., 2021; Zhao et al., 2022; Zhong et al., 2022), genome-wide identification and functional analysis of *PEBP* genes in *H. coronarium* remain unexplored.

This study identified 14 *PEBP* genes in *H. coronarium*, designated *HcPEBP1-14* (**Supplementary Table S3**). The number of *PEBP* genes in *H. coronarium* is fewer than in rice (20) and maize (25), similar to wheat (*Triticum urartu*) (16), but greater than in *Arabidopsis* (6). The variation in *PEBP* gene numbers across species suggests evolutionary differences, with monocotyledons generally having more members than dicotyledons, possibly due to the elimination of non-functional *PEBP* genes during evolution (Yang et al., 2019). Phylogenetic analysis classified 14 *HcPEBP* genes into three subfamilies: 1 MFT, 4 TFL1-likes, and 9 FT-likes (**Figure 2**), consistent with other plant species (Karlgren et al., 2011). Most *HcPEBP* genes clustered with *PEBP* genes from rice, reflecting their monocot status and aligning with previous studies (Yang et al., 2019). The number of *FT* genes and *TFL1* genes is influenced by gene duplication, selective pressures, functional specialization, ecological adaptation, evolutionary trade-offs, and species-specific factors. These include gene duplication events, differential selective pressures, functional diversification, and the need for precise regulation of flowering time in response to environmental changes (Hedman et al., 2009; Pin and Nilsson, 2012; Wickland and Hanzawa, 2015; Andrés et al., 2012). In *H. coronarium* (ginger lily), the FT subfamily has more members than the TFL1 subfamily, which may reflect adaptations to environmental conditions and the need for reproductive success.

Chromosome localization analysis revealed that 9 *HcPEBP* genes are distributed across six chromosomes, while five are located on genomic scaffolds (**Figure 1A**). This distribution may reflect challenges in *H. coronarium* genome assembly. Gene duplication, a common evolutionary mechanism, enhances plant adaptability. Gene duplications include whole-genome and single-gene duplications, with the latter occurring through various mechanisms such as tandem, proximate, diffuse, and separated duplications (Lynch and Conery, 2000; Panchy et al., 2016; Qiao et al., 2019). Intraspecific collinearity analysis identified three pairs of duplicated *HcPEBP* genes (**Figure 1**), suggesting functional similarities. Interspecific collinearity analysis with rice, wild banana, and pineapple revealed homologous relationships among PEBP family members, with *HcPEBP4* and *HcPEBP10* showing collinearity with these species (**Figure 1B**; **Supplementary Table S5**), highlighting their potential significance in PEBP gene family evolution and function.

Conserved motif GxHR and DPDxP are critical for their biological function (Chen et al., 2022). Mutations in or near these regions may affect the interaction of FT protein with FD protein by altering the binding with phosphate ions (Si et al., 2018). All HcPEBPs possessed GxHR motif. Except HcPEBP7, all HcPEBPs contained the DPDxP motif, indicating evolutionary conservation. The absence of the DPDxP motif in HcPEBP7 may lead to the loss of its function. The xGxGGR motif in FT-like proteins and the TAARRR motif in TFL1-like proteins are key distinguishing features that determine their functional roles in flowering regulation. Specifically, FT-like proteins contain the xGxGGR motif, which is essential for their role in promoting flowering. TFL1-like proteins possess the TAARRR motif, which is critical for their function in repressing flowering. This difference in motifs underpins the antagonistic roles of FT and TFL1 proteins in controlling flowering time (Karlgren et al., 2011; Hanzawa et al., 2005; Wickland and Hanzawa, 2015). In *Arabidopsis*, PEBP homologues *AtFT* and *AtTFL1* are key flowering regulators with opposing functions: *AtFT* promotes, while *AtTFL1* represses flowering. Amino acid swaps from Tyr85 and Gln140 in AtFT to His88 and Asp144 in *AtTFL1* reverse their regulatory roles in flowering (Ahn et al., 2006; Hanzawa et al., 2005). Tyr85 in HcPEBP4/9/14 (FT-likes) are mutated to His/Phe/His, this may cause them to lose their function in promoting flowering. The fourth exon’s amino acid residues, divided into four segments, determine gene function specificity, with segments II and III (LYN motif) being crucial for AtFT-induced flowering (Ahn et al., 2006). Among the nine FT-like *HcPEBP* genes, only *HcPEBP4*, *HcPEBP11*, and *HcPEBP14* possess the LYN motif in segment III (**Figure 3**). Gene structure analysis indicates that the number of exons in *HcPEBP* genes ranges from two to five, with most having four exons (**Figure 4D**), a pattern observed in PEBP gene families of other species (Dong et al., 2020; Sun et al., 2023; Wang et al., 2019).

The core promoter and associated *cis*-acting elements are vital for gene transcription regulation, serving as specific protein binding sites (Burley and Roeder, 1996; Molina and Grotewold, 2005; Zou et al., 2011). Light, a key environmental stimulus, influences plant growth and development (Song et al., 2016), with the photoperiod pathway significantly affecting flowering by activating *FT* expression. Elements such as G-box, I-box, and GT1-motif, crucial for light response, are prevalent in the promoters of light-regulated genes (Giuliano et al., 1988; Menkens et al., 1995). The HcPEBP gene family is rich in light-responsive elements, suggesting their role in light-mediated functions. Additionally, *HcPEBP* genes contribute to plant growth, development, stress response, and hormone regulation. Yet, the specific functions of the PEBP gene family *cis*-acting elements in *H. coronarium* have not been studied.

To date, the tissue-specific expression of *HcPEBP* genes in *H. coronarium* remains unexamined. qRT-PCR analysis revealed distinct expression patterns (**Figures 6**, **7**). In soybean and cotton, MFT genes, predominantly expressed in seeds, are implicated in oil content and germination (Cai et al., 2023; Yu et al., 2019). In *Arabidopsis*, *TFL1* expression in shoot meristems prolonged vegetative and inflorescence phases when overexpressed (Fernandez-Nohales et al., 2014). Our study found that the MFT-like gene *HcPEBP8* and the TFL1-like gene *HcPEBP7* are highly expressed in roots and rhizomes, respectively (**Figures 6E–H, M, O, P**), indicating their potential roles in *H. coronarium* root development. Previous research has shown that the florigen encoded by the *FT* gene is produced in leaves and then transferred to the shoot apical meristem, initiating the transition to the reproductive phase (Abe et al., 2005; Susila et al., 2021; Tamaki et al., 2007; Wigge et al., 2005a). In this study, nine *PEBP* genes belong to the FT subfamily (**Figure 2**), with seven exhibiting the highest expression in leaves (**Figures 6A, C–D, I, J, L, N**). The FT-like gene *HcPEBP11*, highly expressed in the inflorescence bud, may influence flower differentiation and development (**Figure 6K**). *OsFT-L1*, expressed at the shoot apical meristem, encodes a florigen-like protein with strong florigenic activity, and its overexpression induces flowering (Giaume et al., 2023). Similarly, *HcPEBP11* is highly expressed in the inflorescence bud, aligning with the expression pattern of *OsFTL1*. Phylogenetic analysis groups *HcPEBP11* and *OsFTL1* together (**Figure 2**). Overexpressing *HcPEBP11* in tobacco confirms its role in promoting flowering, with transgenic lines flowering 9–11 days earlier than wild types. Overexpression of *FT* genes has been shown to accelerate floral organ development and flowering, as seen in cassava with the endogenous FT-like gene *MeFT1* (Odipio et al., 2020). The potential regulatory mechanisms of *HcPEBP11* require further investigation. We hypothesize that:1) The high expression of *HcPEBP11* in floral buds suggests that it may directly function in the floral meristem, independent of long-distance transport from leaves to the shoot apex. 2) It may interact with specific transcription factors (Such as *FD*, *MADS-box* genes) in the floral meristem to directly activate the expression of downstream flowering-related genes. In this study’s findings clarify the number, bioinformatics features, and expression patterns of the PEBP gene family in *H. coronarium*, providing a basis for future research on the role of *HcPEBP11* in regulating flowering.

## 5 Conclusion

In *H. coronarium*, 14 *PEBP* genes were identified and categorized into three subfamilies:9 FT-likes, 4 TFL1-likes, and 1 MFT. The study analyzed their physicochemical properties, phylogeny, gene structure, conserved motifs, and *cis*-acting elements. All HcPEBP proteins share the conserved GxHR motif, and all except HcPEBP7 contain the DPDxP motif. Promoter regions of *HcPEBP* genes are enriched with light-responsive elements. Organ-specific expression analysis via RT-qPCR revealed that *HcPEBP1/2/3/4/9/10/12/14* are highly expressed in leaves, and with increased expression during the transition from vegetative to reproductive growth. *HcPEBP11* shows the highest expression in inflorescence buds, increasing with bud development. Overexpression of *HcPEBP11* in transgenic *tobacco* resulted in early flowering, suggesting its role in flowering regulation. This study provides a comprehensive overview of the PEBP gene family in *H. coronarium* and lays the groundwork for further research into the functional and regulatory mechanisms of *HcPEBP* genes in flowering.

## Data Availability

The original contributions presented in the study are included in the article/**Supplementary Material**. Further inquiries can be directed to the corresponding author/s.
