# Supplementary material for: Genome-wide identification of PEBP gene family in Hedychium coronarium
Source: Front Plant Sci. 2025 Jul 9;16:1482764. doi: 10.3389/fpls.2025.1482764 (PMC12283651; doi:10.3389/fpls.2025.1482764)
Supplement: Supplementary file 1 [file DataSheet1.docx]

Supplementary Material

# Supplementary Data

Supplementary materials for this article can be found in the following file (Supplementary Tables S1 to S9 and Figures S1 to S4).

Supplementary Table S1 List protein accession numbers of the sequences used in phylogenetic tree.

Supplementary Table S2 List of primer sequences in this study.

Supplementary Table S3 HcPEBP gene information.

Supplementary Table S4 The Ka/Ks ratios and estimated divergence time for segmentally and tandemly duplicated *HcPEBP* genes.

Supplementary Table S5 Collinearity relationship of *HcPEBP* genes with *Musa balbisiana*, *Ananas comosus*, *Oryza sativa*

Supplementary Table S6 Sequences information of 8 conserved motifs of HcPEBPs.

Supplementary Table S7 List of cis-regulatory elements elements in the promoter region of *HcPEBP* genes (2000bp upstream of the initiation codon).

Supplementary Table S8 Flowering traits of transgenic tobacco and wild-type.

Supplementary Table S9 FPKM value of HcPEBP genes in four development stages of inflorescence.

Figure S1. The morphological characteristics of root, rhizome, leaf bud, leaf, inflorescence bud.

Figure S2. FPKM (Fragments Per Kilobase of exon model per Million mapped fragments) of *HcPEBP* genes in leaf buds and three development stages of inflorescence buds.

Figure S3. Polymerase chain reaction (PCR) analysis of transgenic tobacco strains.

Figure S4. Subcellular localization of HcPEBP11 proteins in leaf epidermal cells of *Nicotiana benthamiana*.

# Supplementary Figures and Tables

## Supplementary Figures


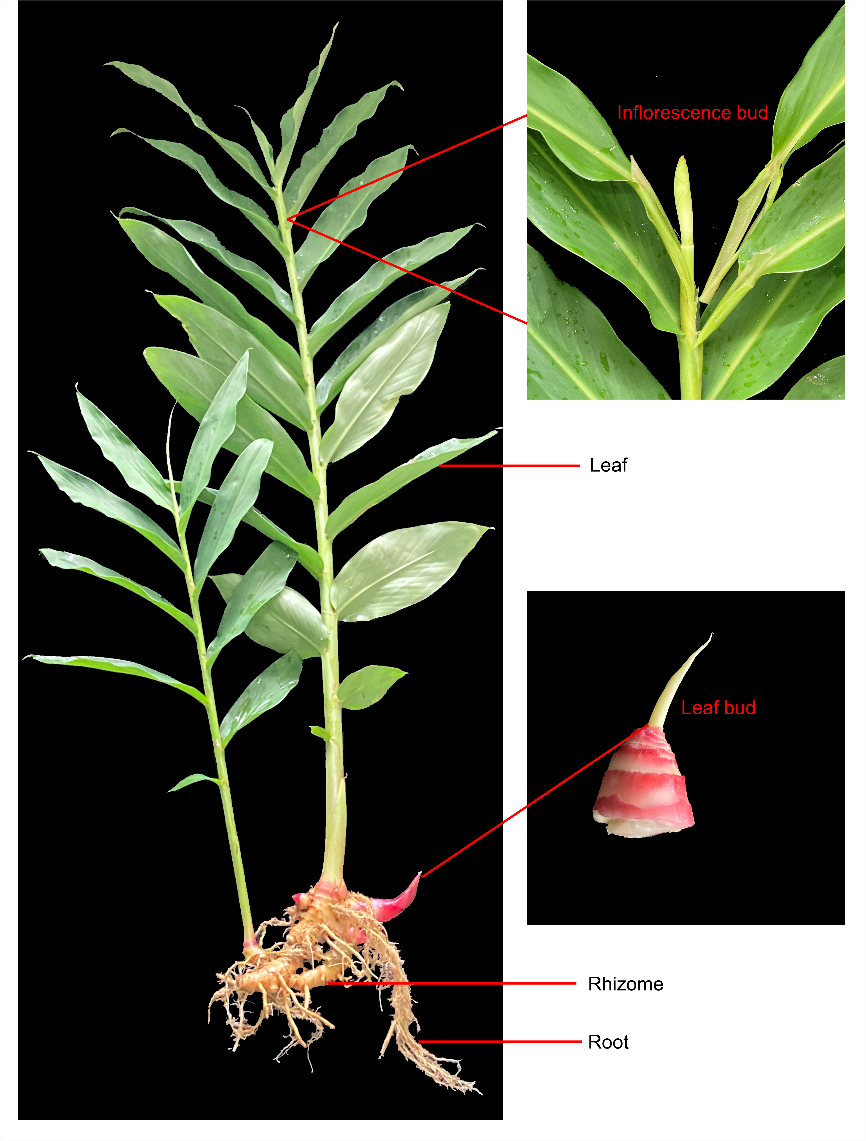


Figure S1. The morphological characteristics of root, rhizome, leaf bud, leaf, inflorescence bud.


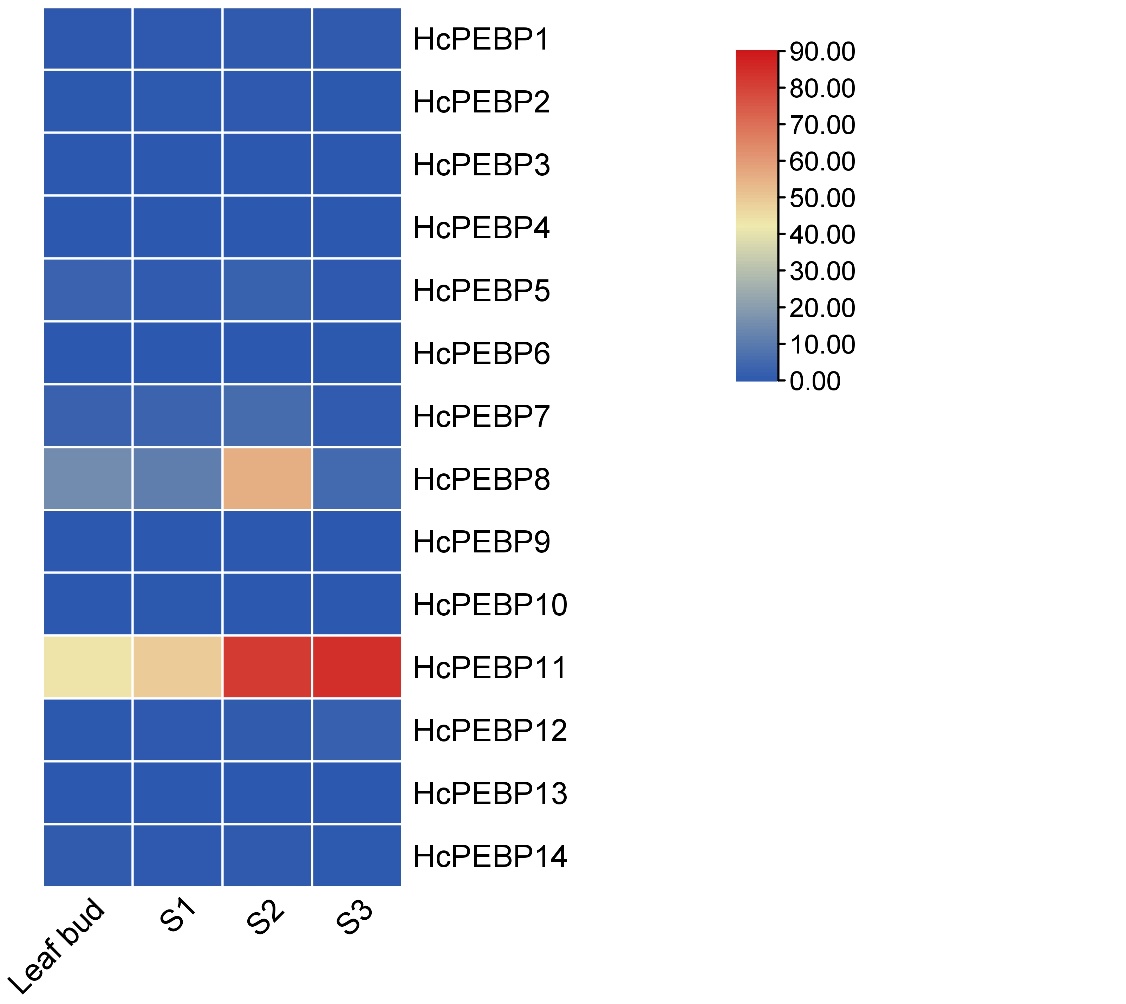


Figure S2. FPKM (Fragments Per Kilobase of exon model per Million mapped fragments) of *HcPEBP* genes in leaf buds and three development stages of inflorescence buds. S1, early stage of inflorescence bud differentiation. S2. middle stage of inflorescence bud differentiation. S3. Late stage of inflorescence bud differentiation.


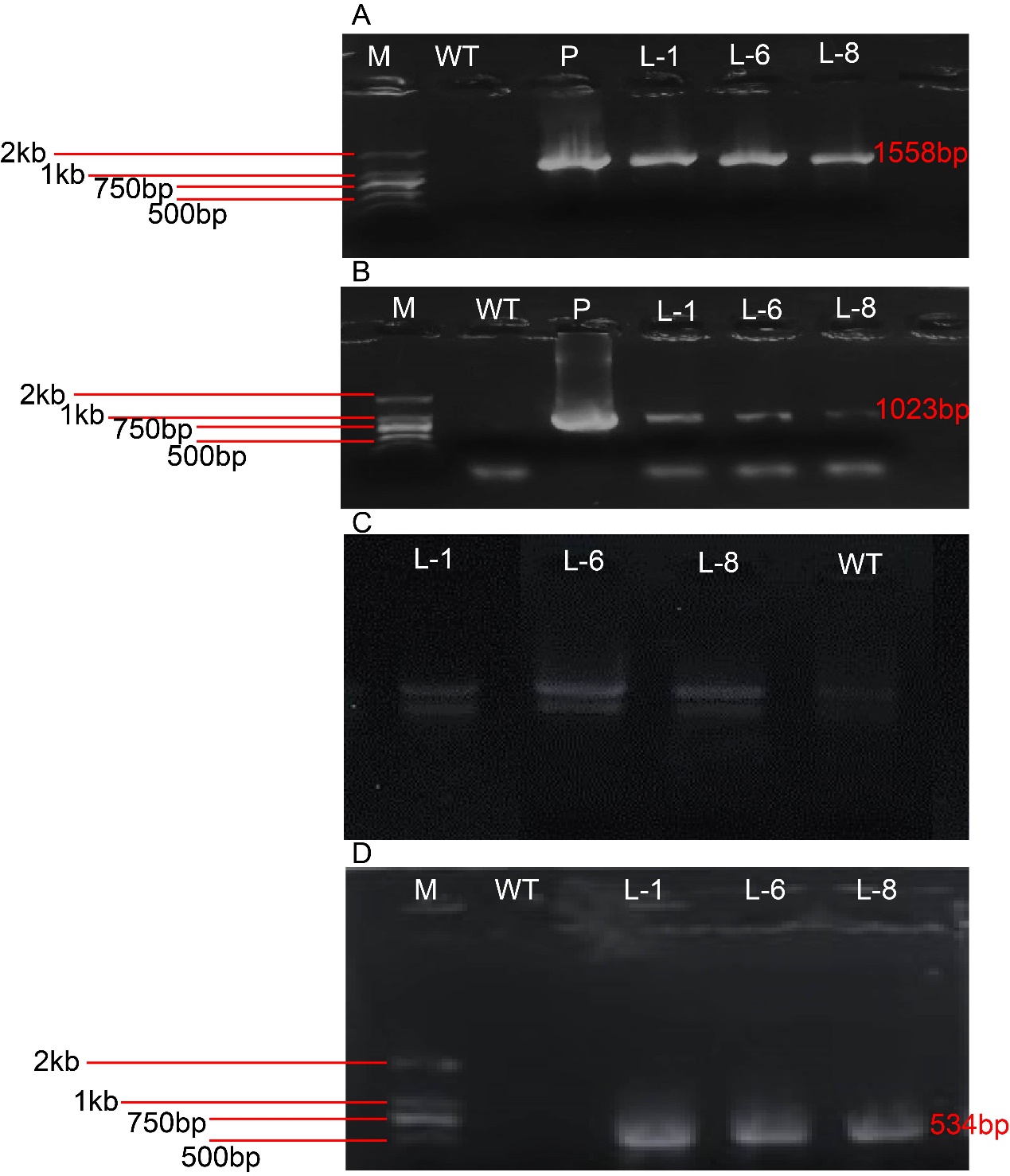


Figure S3 Polymerase chain reaction (PCR) analysis of transgenic tobacco strains. (A) PCR amplification results from three *HcPEBP11* transgenic tobacco lines by universal primer pOx-F/R. (B) PCR amplification results from three *HcPEBP11* transgenic tobacco lines by marker gene-specific primers HPH-F/R. (C) Total RNA of 3 lines for *HcPEBP11* transgenic tobacco and WT tobacco. (D) Semi-quantitative detection of *pOx-HcPEBP11* transgenic tobacco.


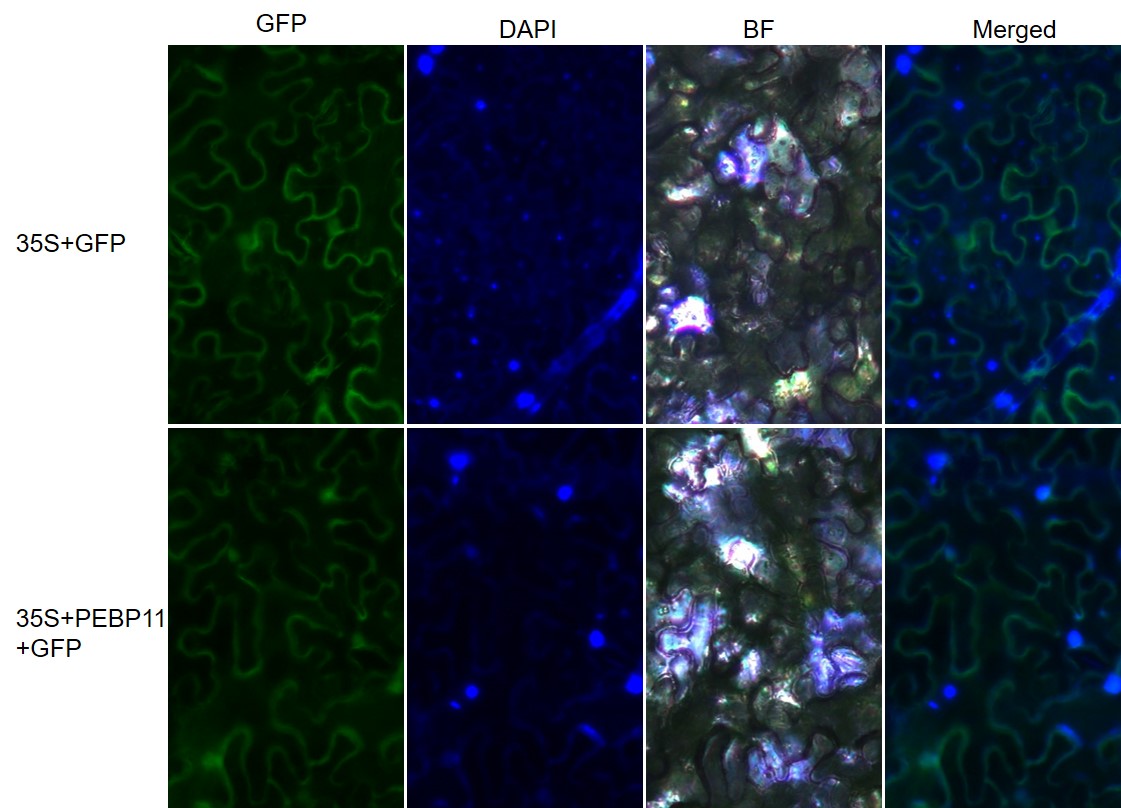


Figure S4 Subcellular localization of HcPEBP11 proteins in leaf epidermal cells of *Nicotiana benthamiana*. The image columns from left to right indicate GFP fluorescence, 4′,6-diamidino-2-phenylindole (DAPI) staining, bright field (BF), and their merged images.
